# Design of a 5G Sub-6 GHz Vehicular Cellular Antenna Element with Consistent Radiation Pattern Using Characteristic Mode Analysis

**DOI:** 10.3390/s22228862

**Published:** 2022-11-16

**Authors:** Ehab Abdul-Rahman, Daniel N. Aloi

**Affiliations:** Electrical and Computer Engineering Department, Oakland University, Rochester, MI 48309, USA

**Keywords:** 5G sub-6 GHz, automotive antenna design, characteristic mode analysis, UWB, radiation pattern

## Abstract

A cellular 5G sub-6 GHz vehicle antenna design with a consistent radiation pattern across the frequency bands in 0.617–5 GHz is demonstrated via characteristic mode analysis. The design focuses on maintaining monopole first-order mode radiation pattern over cellular frequency bands and avoiding higher-order modes out of the operational frequency bands to provide optimal performance for automotive requirements. Rather than using an empirical design method, the design procedure in this paper uses the calculated modal significance, characteristic current, modal radiation pattern, and reflection coefficient to define the antenna structure dimensions. The proposed design was simulated, a prototype was measured, and the performance was evaluated on a 1-m ground plane. The antenna has perfect omnidirectionality with a high and stable gain across the frequency range in the 30° area above the horizon.

## 1. Introduction

The concept of a connected vehicle with high throughput internet is becoming necessary as it allows extra functional and entertainment services [1]. Long-term evolution (LTE) technologies and 5G enhancements provide a solution for high-speed and low latency communication channels. New frequency bands were defined and classified in the sub-6 GHz frequency ranges from 0.617–0.96 GHz, 1.71–2.7 GHz, and 3.3–5.0 GHz [2]. This new wide frequency range introduces challenges for antenna design

Vehicle cellular antennas must have an omnidirectional radiation pattern and gain maximized at lower elevations for optimal performance. However, designing a wideband monopole with a consistent radiation pattern across the entire frequency range encounters a challenge due to excited higher-order radiation modes, which affect the elevation pattern, or asymmetrical designs, which affect the azimuth pattern.

The ultra-wideband (UWB) antenna structures with one consistent radiation pattern for all frequencies are known as frequency-independent structures [3]. Conical antennas are one of those structures that would provide a radiation pattern that meets automotive requirements as in [4]; however, the size and shape of this structure are difficult to implement on vehicles due to styling constraints. Other UWB solutions include the planar wideband structure in [5,6]; the elevation pattern has low gain at low elevation angles in frequencies around 2.5 GHz and 4 GHz, respectively, due to the interruption of the higher-order modes. Multi-branch design concepts, as in [7,8], would improve the gain at lower elevations. However, due to its non-symmetrical design and blockage from longer branches, the azimuth pattern loses omnidirectionality at higher frequencies. Automotive antenna designs [9,10,11] only consider LTE 4G bands covering only 700–960 MHz and 1700–2700 MHz.

Recent antennas designed for 5G sub-6 GHz bands fit the shark-fin style [12,13,14,15]. Still, none of those have discussed optimal radiation patterns for both the azimuth plane (H-plane) and the elevation plane (E-plane). In [12], directionality is degraded for frequencies higher than 2.1 GHz. In [13], the antenna exhibits good omnidirectionality; however, it has low gain at lower elevations, as shown from the plots for 2.64 GHz and 3.42 GHz. In [14], the linear average gain (LAG) has a gain drop in the frequency range between 2.4–3.8 GHz and degradation in omnidirectionality for frequencies beyond 3.9 GHz. Similarly, for the antenna design in [15], the radiation pattern loses directionality at 3.3–5 GHz, and its LAG decreases in the frequency range between 4.2–5 GHz. The proposed antenna resolves those radiation pattern defects by utilizing the modal analysis to synthesize the intrinsic monopole first-order mode radiation pattern for all operating bands and avoid higher-order modes interrupting the in-band performance. The results show more consistent gain and omnidirectionality across the desired frequency ranges that better match automotive requirements.

### 1.1. Introduction to the Theory of Characteristic Modes

The theory of characteristic modes (TCM) gives a direct insight into the radiating phenomena occurring on the antenna. It was first proposed by *Garbacz and Turpin* [16] in 1971. The computation was simplified by *Harrington’s* solving the eigenvalue equation for the current and tangential electric field on the metal body [17,18]. Nowadays, it is widely used for different aspects of antenna design, such as increasing the impedance bandwidth by changing the structure, source location, or feed network [19,20,21,22,23]. It can also be utilized to enhance the isolation between antenna elements in a MIMO system, mainly for handheld applications [24,25,26]. Other uses of this technique are beneficial for radiation pattern steering [27,28,29] and radiation pattern enhancements [30,31,32,33,34] over narrow frequency ranges. UWB planer monopole designs were analyzed using the TCM in [35,36]. However, these studies focused on the frequency bandwidth and the effect of adding a slot on the structure; the studies did not address radiation pattern improvements. In contrast, the contribution of this manuscript uses the TCM to avoid higher-order modes and provide a consistent radiation pattern for the UWB 5G cellular antenna for automotive applications. 

When a radiating metal structure is immersed in a propagating electromagnetic field, it introduces a tangential current on the surface of the metallic body as represented in Equation (1), where the *L* operator represents an integral equation that relates the tangential current to the electric field [17].

(1) $${\left[L\left(J\right)-E\right]}_{tan}=0$$

The *L* operator has a dimension of impedance R + jΧ, where R and X are the real and imaginary impedance of the tangential metal body, *J* is the surface current, *E* is the electric field and the null on the right-hand side of the equation indicates the absence of the excitation source. The TCM defines an orthogonal set of current modes ($J_{n}$) by solving the scattering matrix eigenvalue and eigenvectors for Equations (2) and (3).

(2) $$\left(R+\mathrm{jX}\right)\left(J_{n}\right)=v_{n}R\left(J_{n}\right)$$

(3) $$v_{n}=1+j\lambda_{n}$$

where $\lambda_{n}$,$\;v_{n}\;$ are the eigenvalue and the eigenvector. The total surface current *J* can be represented as a weighted sum $\sum \alpha_{n\;}J_{n}$. Each $J_{n}\;$ represents the exciting current for mode (*n*) that can be isolated and tracked over frequency. The main parameters used in the modal analysis are eigenvalue, modal significance, characteristic angle, and modal weighting coefficients [37].

### 1.2. Automotive Requirements and Constraints

**Figure 1:**
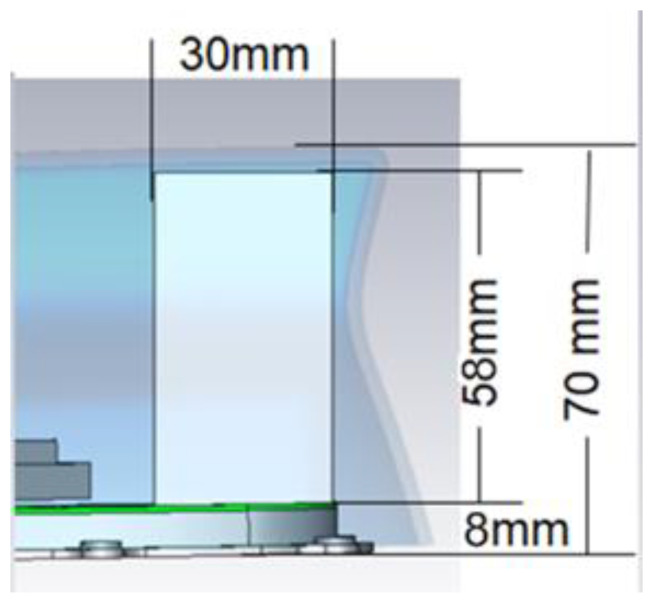
Vehicle shark fin rear-section to illustrate antenna size constraint.

The rooftop shark fin’s total height is constrained to 70 mm. As shown in Figure 1, the plastic cover thickness and clearance to the element is approximately 4 mm from the top and a mounting base of 8 mm from the bottom. This limits the designer’s space to an element height of around 58 mm and up to 30–40 mm width to fit other antenna element functions such as GNSS and SXM. 5G cellular antennas are part multiple input multiple output (MIMO) system [38]. Cellular elements can co-exist in one shark fin if the styling allows; otherwise, antennas may be distributed in other locations in the vehicles. MIMO helps mitigate channel fading and multipath signal fluctuation, mainly when operating at high frequencies. 

**Figure 2:**
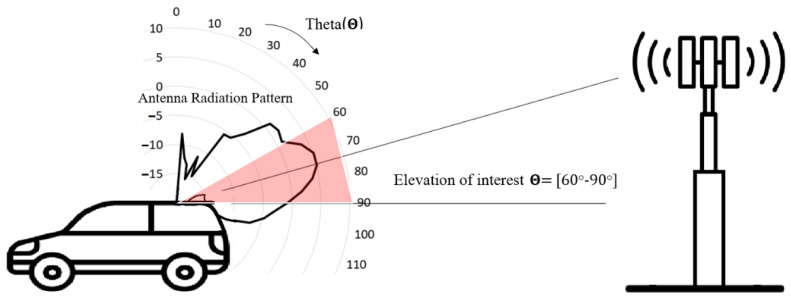
Automotive radiation pattern requirement relative to cell tower.

Automotive cellular antenna element radiation patterns must be omnidirectional since the vehicle can be in any heading direction relative to the cell tower. The elevation, radiation pattern, and gain need to be maximized toward the horizon to enhance the communication link when the vehicle is farther away from the cell tower, as illustrated in Figure 2. In addition, considering land terrain of ±10 degrees and elevation spread angle from the channel model [39], the antenna gain needs to be maximized for theta angles between theta = 60–90 degrees.

It is common for automotive antennas to be evaluated in terms of LAG at each frequency and elevation range, as calculated via Equation (4), and to determine the omnidirectionality of the antenna, the gain standard deviation (STD) metric is commonly used, and it is calculated over the 360-degree azimuth via Equation (5).

(4) $$LAG{\left(f,\theta \right)}_{dB}=10\log_{10}\frac{\sum_{1}^{N\emptyset }G_{lin}\left(f,\theta ,\emptyset \right)}{N\emptyset }\;$$

(5) $$STD{\left(f,\theta \right)}_{dB}=\sqrt[{2}]{\frac{\sum_{1}^{N\emptyset }{\left(G_{dB}\left(f,\theta ,\emptyset \right)-\bar{G_{dB}\left(f,\theta \right)}\right)}^{2}}{N\emptyset }}$$

where *N*∅ is the number of gain measurement points over the 360 degrees azimuth (∅) for fixed frequency (*f*) and elevation (*θ*), $G_{lin}$ is gain in linear scale, and $G_{dB}$ is gain in *dB* scale.

### 1.3. Paper Organization

The paper is organized as follows: the Material and Methods section describes the proposed antenna design and its characteristic mode analysis that shows desired versus undesired radiation modes for automotive applications. It also shows the modal tuning method and the parametric study using modal significance to control the mode bandwidth and resonance frequency. Next, the Results section discusses the antenna performance for the simulation data and the measured prototype in terms of VSWR, 2D radiation pattern, efficiency, LAG, and STD. Finally, the last section provides the conclusions of this study.

## 2. Materials and Methods

### 2.1. Design Description

**Figure 3:**
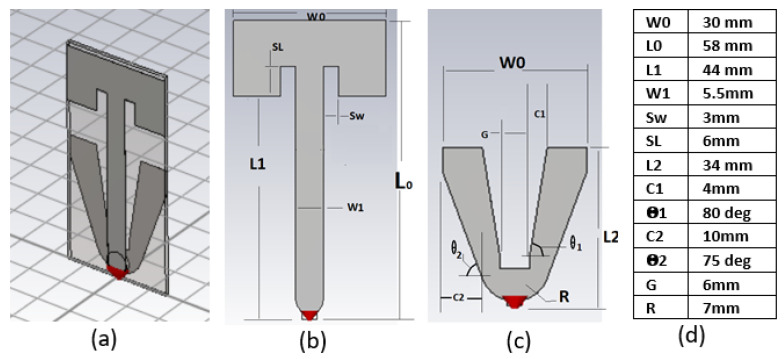
Proposed antenna structure: (**a**) whole structure, (**b**) long-structure, (**c**) short-structure, and (**d**) table of dimensions.

The idea of the proposed design is to use two structures: long-structure and short-structure; each covers a portion of the frequency range with a first-order mode as a desired radiation pattern mode for automotive applications. The antenna is built on a two-layer FR4 printed circuit board (PCB) with a relative dielectric constant of 4.3. Each structure is printed on one layer and connected at the feeding point, as illustrated in Figure 3a; the dimensions are shown in Figure 3d. The antenna is intended to be symmetric around the centerline to maintain the roundness of the omnidirectional radiation pattern for the high-frequency bands.

The first structure top layer, “long-structure”, Figure 3b, is a T-shaped planar monopole structure to utilize the height available to cover the low-frequency range of 0.617–0.96 GHz. The second property of this structure is it controls higher-order modes with undesirable radiation patterns in the non-cellular frequency range from 2.7–3.3 GHz and beyond 5 GHz, respectively.

The second structure, “short-structure”, Figure 3c, is a wideband planar monopole intended to cover the frequency range from 1.71–5 GHz with a first-order frequency mode and to minimize the influence of higher-order modes prior to 5 GHz by reducing its modal significance. The structure has a trapezoidal gap in the centerline to minimize the coupling to the long-structure. In addition, the gap size helps to provide high impedance at the global navigation satellite system (GNSS) frequency band to offer a decent noise rejection and avoid desensitization for possible adjacent GNSS function. The detailed design parameters are discussed in the following section.

The design dimensions were selected based on characteristic mode analysis, which allows for synthesizing and controlling the dominant radiation mode for each frequency range, as described in the following two sections.

### 2.2. Antenna Characteristic Mode Analysis

**Figure 4:**
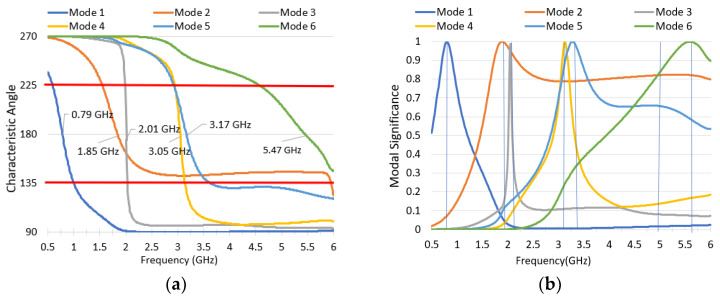
Characteristic mode analysis for the proposed antenna: (**a**) modal characteristic angle and (**b**) modal significance.

By running the CMA for the complete antenna structure over an infinite ground plane using CST simulation software [40], the characteristic angle and modal significance for the first six excited modes are shown in Figure 4a,b, respectively. Mode 1 resonance is at 790 MHz and shows good potential to cover the low band frequency range from 617–960 MHz. The Mode 2 resonance is at 1850 MHz and has potential bandwidth extending from 1.7 to 5 GHz. Mode 3 resonates at 2.01 GHz. Mode 4 and Mode 5 have resonances at 3.05 and 3.17 GHz, respectively. Finally, Mode 6 resonates at 5.47 GHz; it takes over Mode 2 and starts to dominate the radiation performance beyond the 5 GHz.

**Figure 5:**
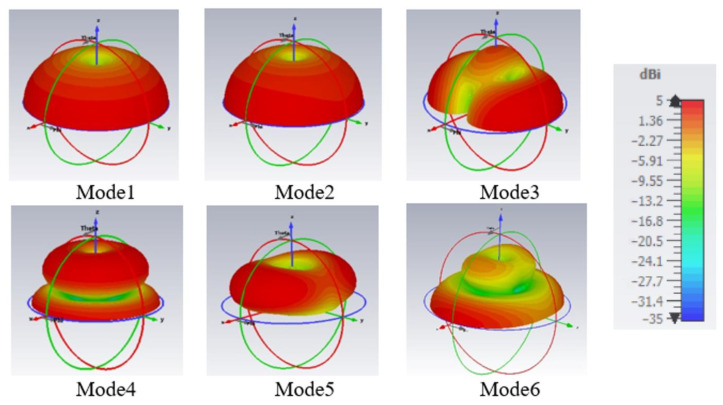
Modal radiation pattern (directivity) at resonance frequencies for Mode 1 at 0.79 GHz, Mode 2 at 1.85 GHz, Mode 3 at 2.01 GHz, Mode 4 at 3.05 GHz, Mode 5 at 3.17 GHz, and Mode 6 at 5.47 GHz.

**Figure 6:**
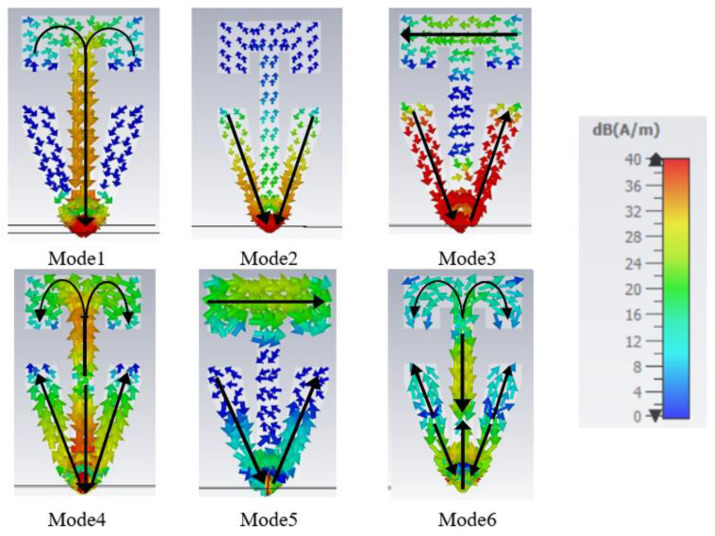
Modal current distribution at resonance frequencies for Mode 1 at 0.79 GHz, Mode 2 at 1.85 GHz, Mode 3 at 2.01 GHz, Mode 4 at 3.05 GHz, Mode 5 at 3.17 GHz, and Mode 6 at 5.47 GHz.

The radiation pattern and the current flow for each characteristic mode are shown in Figure 5 and Figure 6, respectively. Mode 1 is a first-order mode excited by the long-structure. Mode 2 is also a first-order mode that is excited from the short-structure. Both Mode 1 and Mode 2 have the desired radiation pattern for automotive applications since they have high gain at the horizon. Mode 3 exists because of the trapezoidal cut in the short-structure, which allows the currents to travel parallel to the ground plane; similarly, Mode 5 has current traveling horizontally across the top of the long-structure. Mode 3 and Mode 5 are not excited with the intended application when the feeding point is at the bottom of the structure. Mode 4 is a combined second-order mode of the long-structure and first-order mode of the short-structure. Its radiation pattern has a null at low elevations, making it unsuitable for the automotive requirement. Thus, the structure was tuned to narrow bandwidth and has low modal significance in the cellular bands, as is discussed in the next section. Mode 6 combines the third-order mode for the long-structure and the second-order mode for short-structure; the radiation pattern has an elliptical shape in the azimuth plane and a second beam upward in the elevation plane. Since both Mode 4 and Mode 6 interrupt the operation of Mode 2, causing undesired radiation patterns, it is vital to tune those out of the operational frequency range of 2.7–3.3 GHz or beyond 5 GHz.

### 2.3. Optimization Method and Parametric Study

Using the CMA tool, modal significance was monitored while changing the structure dimension to set each mode on the intended frequency range as shown in the previous section, where the desired mode has high modal significance over cellular bands, and undesired modes have low modal significant value in the cellular frequency ranges.

#### 2.3.1. Long-Structure Tuning

**Figure 7:**
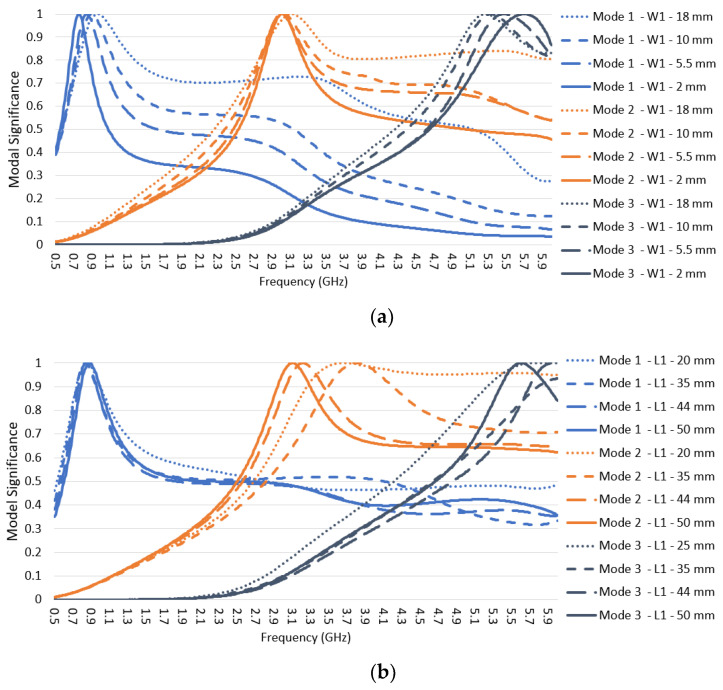

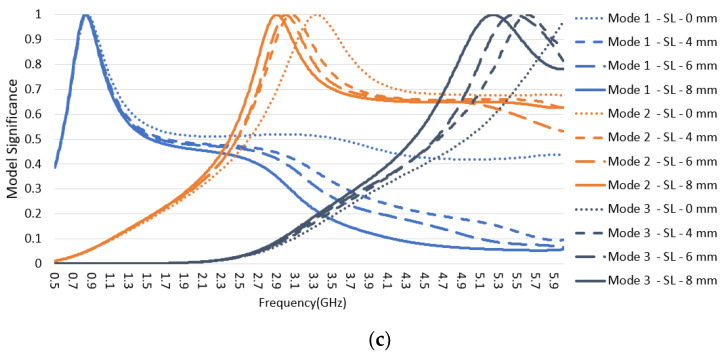
Modal significance for the long-structure while varying three dimensions in Figure 3: (**a**) vertical width W1, (**b**) vertical height L1, and (**c**) slot SL.

The long-structure has three radiation modes, shown in Figure 7: Mode 1, Mode 2, and Mode 3 correspond to Mode 1, Mode 4, and part of Mode 6 in the whole structure characteristic mode analysis discussed in Section 2.2. Mode 1 is intended to have a first-order mode in the frequency range from 617–960 MHz, Mode 2 and Mode 3 are higher-order modes that need to be avoided in cellular bands in the frequency range of 2.7–3.3 GHz and beyond 5 GHz, respectively. The modal significance metric was used to adjust each mode’s frequency range while sweeping the three main dimensions of the long-structure W1, L1, and SL, as shown in Figure 3b. 

The width W1 was evaluated at the following values {2, 5.5, 10, 18 mm} for L1 = 44 mm and SL = 6 mm, and the results are shown in Figure 7a. As W1 decreases, the resonance for Mode 1 shifts down to cover lower frequencies, Mode 3′s resonance shifts up in frequency, while Mode 2′s resonance is unchanged. Additionally, the potential bandwidths for Mode 1, Mode 2, and Mode 3 decrease. Therefore, the design value selected was 5.5 mm mainly to maintain sufficient bandwidth of Mode 1 to cover the low-frequency range of 617–960 MHz.

The dimension L1 was evaluated at L1 = {20, 35, 44, 50 mm}, for W1 = 5.5 mm and SL= 0 mm. The corresponding modal significance values are plotted in Figure 7b. As L1 increases, the bandwidth for Mode 2 significantly decreases, and its resonance shifts down toward the intended non-cellular frequency range of 2.7–3.3 GHz. The design value selected was 44 mm, showing a decent narrow bandwidth for Mode 2 and lower modal significance for Mode 3 before 5 GHz. Any higher L1 starts affecting the Mode 1 modal significance at 617 Mhz. Since Mode 2 still has high modal significance at 3.3 GHz, it needs to be further adjusted by tuning the SL slot dimension.

The slot SL was evaluated at SL = {0, 4, 6, 8 mm} for L1 = 44 mm and W1 = 5.5 mm. The modal significance results are plotted in Figure 7c. The SL slot serves as a fine tune and a compromise between Mode 2 and Mode 3. As SL increases, Mode 2 and Mode 3 shift down in frequency, while Mode 1 has a non-significant bandwidth change. The design value was selected as 6 mm to place Mode 2 in the right spot in the middle of the non-operational frequency range of 2.7–3.3 GHz and to stop the lower edges of Mode 2 and Mode 3 from having significant values for frequencies at 2.7 GHz and 5 GHz, respectively. 

#### 2.3.2. Short-Structure Tuning

**Figure 8:**
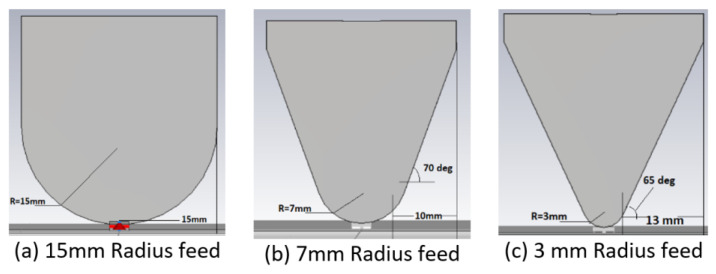
Short-structure feeding width.

Three different feed widths for the short-structure were analyzed as shown in Figure 8. All shapes have a semi-circle feed shape with radii of 15 mm, 7 mm, and 3 mm; also, the three shapes share the outer dimension top widths of 30 mm, and total heights of 34 mm. 

**Figure 9:**
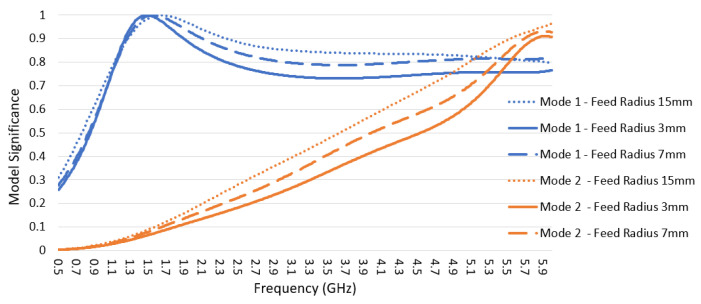
Modal significance for the three feeding sizes is shown in Figure 8.

**Figure 10:**
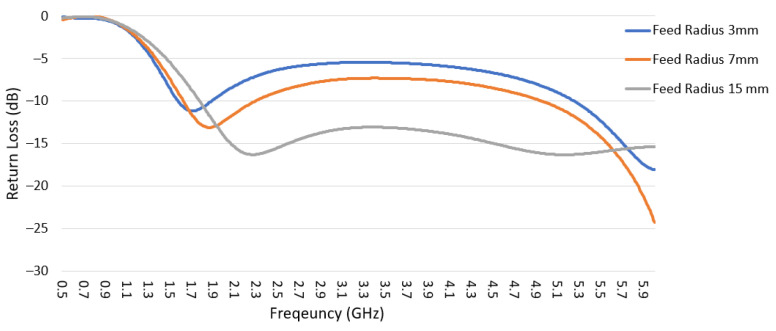
Return loss for the three feeding sizes is shown in Figure 8.

The short-structure has two modes shown in Figure 9: Mode 1 and Mode 2 correspond to Mode 2 and part of Mode 6 in the whole structure characteristic mode analysis discussed in Section 2.2. Mode 1 is intended to cover the frequency range from 1.71 to 5 GHz with the first-order mode, and Mode 2 is a higher-order mode that needs to be avoided in cellular bands and has low modal significance before 5 GHz. The modal significance and return loss data for the three feed sizes are shown in Figure 9 and Figure 10, respectively. As the feed width decreases, Mode 1 resonance frequency barely changes; however, the modal significance value decreases in the frequency range 2.3–5 GHz, which also introduces higher return loss, as shown in Figure 10. Similarly, for Mode 2, as the feed width decreases, the modal significance value decreases for frequencies before 5 GHz, which helps to reduce the effect of this undesired mode. Therefore, the compromised design feed radius selected was 7 mm, to maintain adequate modal significance and return loss below 7 dB for Mode 1 while obtaining lower modal significance for Mode 2.

#### 2.3.3. Combining Long and Short Structures

**Figure 11:**
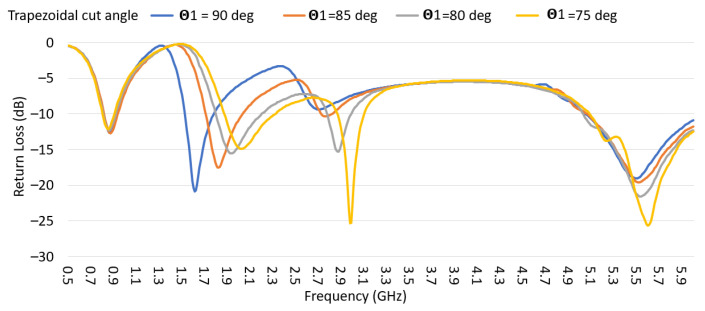
Return loss while sweeping the trapezoidal cut angle (Θ1) in the short structure.

Even though the short and long structures operate on different frequency ranges, the direct connection between the two structures does not give the expected complex admittance sum because the coupling between the two structures generates a mutual impedance and changes the excitation mechanism between the structures. Thus, a trapezoidal cut was introduced in the short-structure to minimize the coupling. The return loss was monitored in Figure 11 with a different value of Θ1 while keeping G = 6 mm, as shown in Figure 3c. As Θ1 increases, the coupling between the long and short structures decreases, and the high band frequency resonance moved toward higher frequencies and achieved a better 50 ohm impedance match in the mid-frequency range. The design value Θ1 was selected to be 80 degrees to maintain a good match at 1710 MHz.

On the other hand, the gap between the long and short structures controls the impedance between the low and high bands giving decent rejection and high impedance at the GNSS frequency bands. For instance, for L1 GNSS frequency range 1.559–1.61 GHz, the return loss is 0.5 dB to 1 dB, which would provide 9.6 dB to 6.8 dB extra rejection for cellular noise into the GNSS band. 

**Figure 12:**
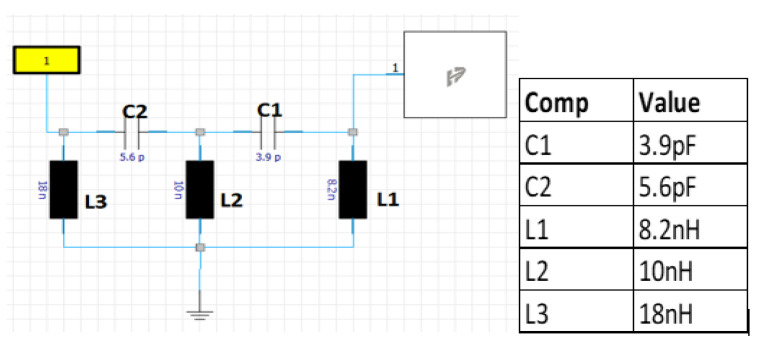
Antenna matching circuit and component values.

Finally, the matching circuit in Figure 12 was introduced to the feeding point where the antenna is connected to the horizontal PCB on the baseplate as shown in Figure 1, Section 1.2. The matching circuit extends the impedance match down to 617 MHz and improves the return loss for the frequency range from 3.3 GHz to 5 GHz.

## 3. Results

### 3.1. Antenna Evaluation Setup

**Figure 13:**
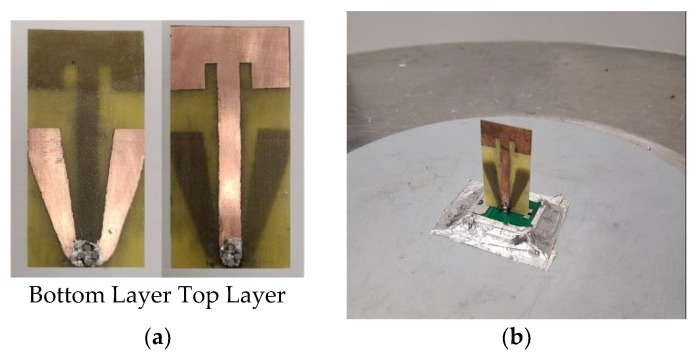

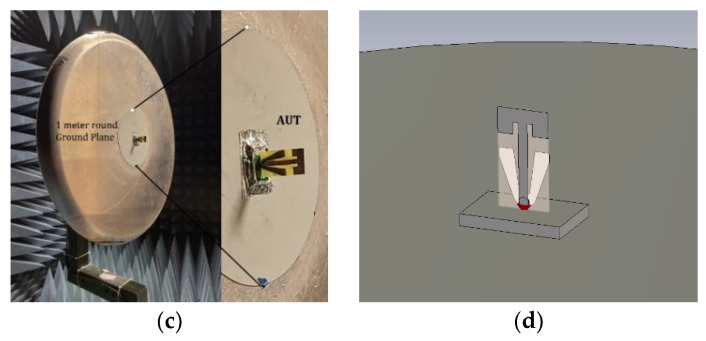
(**a**) Fabricated antenna; (**b**) antenna prototype; (**c**) antenna measurement setup; (**d**) simulation model.

The antenna was fabricated on FR-4 using a PCB milling machine, as shown in Figure 13a. Both top and bottom layers were connected using four vias soldered at the feed point. The overall structure was mounted on a horizontal PCB with the matching circuit populated. The horizontal PCB is connected to a metal baseplate of 8 mm height to mimic the shark-fin environment, as shown in Figure 13b.

The prototype antenna was measured in a far-field anechoic chamber inner dimension of 13 × 4 × 4 m^3^, and the measurement distance between the source antenna and AUT was 10 m; The AUT gain was calculated using the gain substitution method relative to standard antennas.

The AUT was evaluated on a 1-m ground plane, as shown in Figure 13c. The 1-m ground plane is a typical setup to assess an automotive antenna element despite the different vehicle’s roof shapes, tilt, and sizes. The antenna was simulated using CST time-domain solver software [40] with the same setup as the prototype is shown in Figure 13d.

All simulation and measurement data were collected for the full sphere with a resolution of 5 degrees in azimuth and 5 degrees in elevation, and for three frequency ranges: low band 0.617–0.96 GHz in a 20 MHz frequency step, high band 1.71–2.7 GHz in a 50 MHz frequency step, and ultra-high band 3.3–5 GHz with a frequency step of 75 MHz.

### 3.2. VSWR

**Figure 14:**
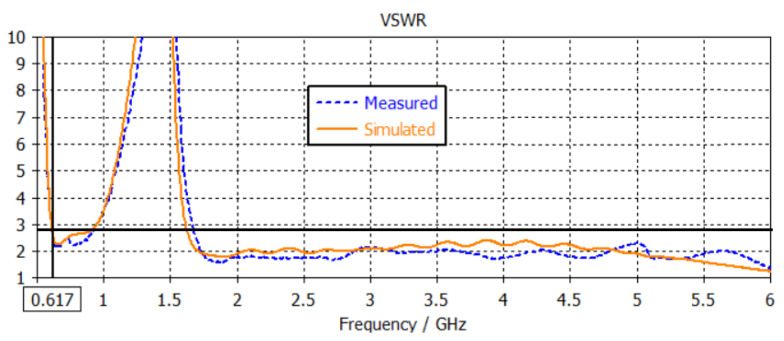
Comparison of measured and simulated VSWR.

The S-parameters of the simulating results, including the matching circuit, were collected. The prototyped antenna was also measured with a similar setup at the feeding point, and the same matching circuit was applied. Both simulated and measured results are plotted in Figure 14. The VSWR was below 2.7:1 for the low-frequency range 617–960 MHz and 2.5:1 for frequencies 1.71–5 GHz.

### 3.3. Radiation Pattern

**Figure 15:**
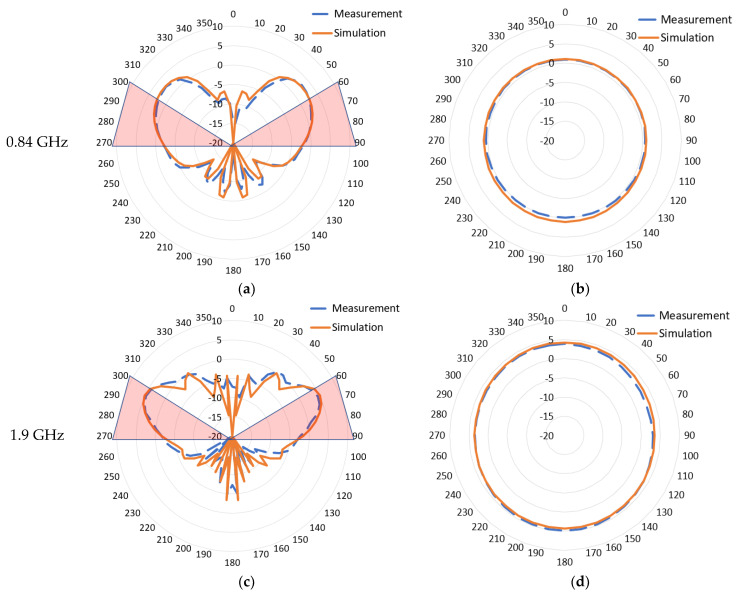

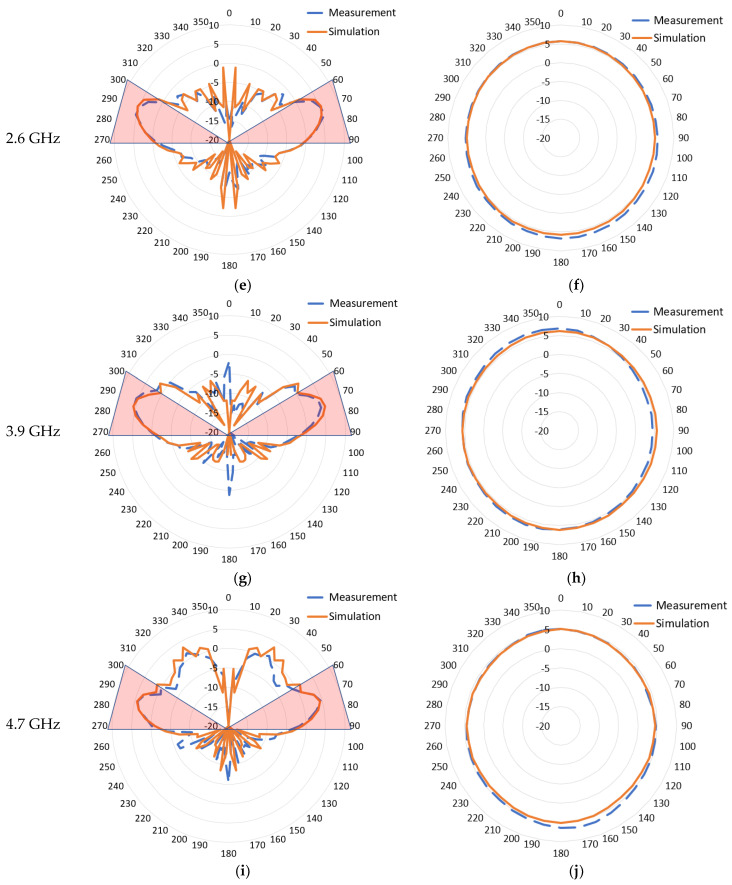
2D Radiation pattern for (**a**) 0.84 GHz elevation plane (ϕ = 90); (**b**) 0.84 GHz azimuth plane (Θ = 75); (**c**) 1.9 GHz elevation plane (ϕ = 90); (**d**) 1.9 GHz azimuth plane (Θ = 75); (**e**) 2.6 GHz elevation plane (ϕ = 90); (**f**) 2.6 GHz azimuth plane (Θ = 75); (**g**) 3.9 GHz elevation plane (ϕ = 90); (**h**) 3.9 GHz azimuth plane (Θ = 75); (**i**) 4.7 GHz elevation plane (ϕ = 90); (**j**) 4.7 GHz azimuth plane (Θ = 75).

This antenna design aims to maintain the intrinsic monopole first-order mode radiation pattern across the new 5G cellular frequency ranges. The 2D radiation pattern for the simulated realized gain compared to the prototype measurements at different frequency points is shown in Figure 15.

The elevation plane radiation pattern at ϕ = 90° for the frequencies 0.84 GHz, 1.9 GHz, 2.6 GHz, 3.9 GHz, and 4.7 GHz are shown in Figure 15a,c,e,g,i, respectively. Both measurement and simulation have a good match. The radiation pattern is generally focused toward the elevation of the interest area shaded in red (theta = [60–90] degrees). Since the antenna was simulated/measured on a finite 1-m ground plane, peak gain tends to move lower elevations as the frequency becomes higher due to the relatively larger electrical size of the ground plane. At 0.840 GHz, the peak gain was 2.7 dBi at theta 50 degrees; however, the beam is broad and covers the intended elevation zone very well. At the mid-frequency ranges 1.9, 2.6, and 3.9 GHz, the beam is very well set in the intended elevation ranges with a peak gain of 5 dBi. At 4.7 GHz, the pattern starts to have a second beam at higher elevations due to the influence of higher-order Mode 6 described in Section 2.2. The main lower beam becomes narrower but still effectively covers the intended range with a peak gain of 4.5 dBi.

The azimuth plane radiation pattern at Θ = 75° for the frequencies 0.84 GHz, 1.9 GHz, 2.6 GHz, 3.9 GHz, and 4.7 GHz are shown in Figure 15b,d,f,h,j, respectively. The measurement results exhibit excellent omnidirectionality throughout the operational frequency bandwidth and a good match to simulation data with a slight drift due to an imperfect prototype build and measurement accuracy. As the frequency grows higher, the radiation pattern shape tends to be slightly elliptical because of the higher-order mode.

### 3.4. Performance across the Frequency Range

**Figure 16:**
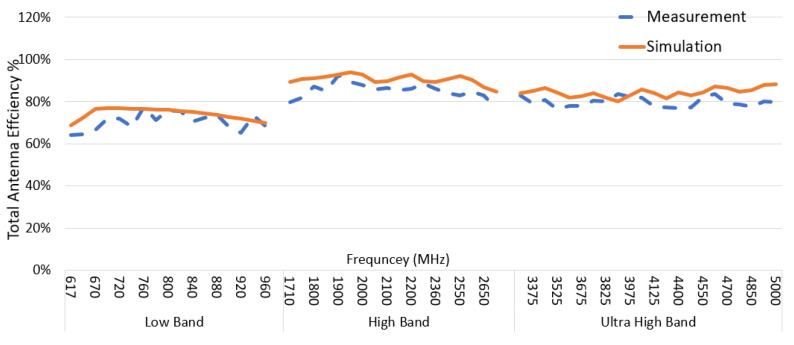
AUT total efficiency for prototype measurement and simulation data.

Antenna total efficiency was collected from simulation software and measurement data, as shown in Figure 16. In both cases, the efficiency values include the matching circuit and mismatch loss. Measurement data has a slightly lower average efficiency of around 5% compared to the simulation data, probably due to a non-ideal matching circuit and actual PCB material loss on the prototype; overall, the trend matches. The average measurement efficiency for low, high, and ultra-high bands are 70%, 85%, and 79%, respectively. Measurement data show more fluctuation over frequency, especially at lower frequencies, possibly due to measurement accuracy in the chamber.

**Figure 17:**
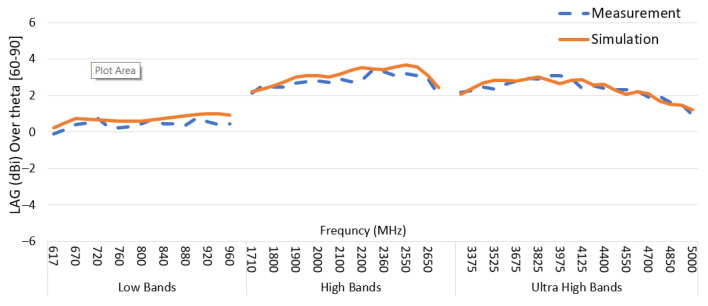
AUT linear average gain for elevation zone theta (60–90°) across the frequency range for prototype measurement and simulation data.

The primary automotive antenna performance metric is the LAG at specified elevation angles. The LAG for theta = [60–90] degrees was calculated by Equation (4) at each frequency point and plotted in Figure 17. Results show a consistent and flat gain across frequency and a similar gain for measurement and simulation. The low band average LAG is between 0 dBi and 0.9 dBi. The high-frequency band’s average LAG is between 2 dBi and 3.5 dBi. Finally, the average LAG for ultra-high bands is between 1.4 dBi and 2.8 dBi.

**Figure 18:**
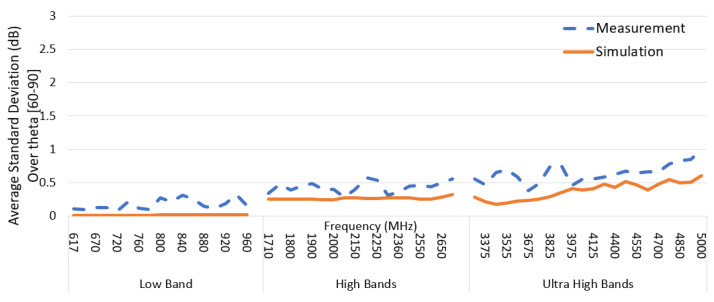
AUT average standard deviation for elevation zone theta (60–90°) across the frequency range for prototype measurement and simulation data.

Similarly, to illustrate the omnidirectionality across the operating frequency range, the average STD for theta = [60–90 degrees] was calculated by Equation (5) and is shown in Figure 18. Again, the trend for both the measured and simulated antenna shows a good correlation. Measurement data is 0.5 dB higher on average due to measurement accuracy. In the ultra-high band, the STD slightly ramps up as the azimuth pattern tends to an elliptical shape, as discussed in Section 3.3 for the azimuth plane plots.

## 4. Conclusions

The article presents an antenna design for the newly defined 5G sub-6 GHz bands for vehicle rooftop applications. The design method utilizes the CMA to avoid higher-order modes and provide an optimized and consistent radiation pattern across the cellular frequency ranges; low band 0.617–0.96 GHz, high band 1.7–2.7 GHz, and ultra-high band 3.3–5 GHz. 

The antenna was fabricated on a two-layer FR4 PCB with a size of 58 × 30 × 1 mm^3^. The performance was evaluated by a prototype measurement and simulation data on a 1-m diameter ground plane. The VSWR was below 2.7:1, the average antenna efficiency was 80%, and the LAG for theta between (60–90 degrees) was 0.5 dBi for frequencies below 1 GHz and 2.5 dBi for frequency bands higher than 1 GHz up to 5 GHz. The gain standard deviation for 360-degree azimuth at theta angles close to the horizon was below 1 dB.
